# Growth and remodelling for profound circular wounds in skin

**DOI:** 10.1007/s10237-014-0609-1

**Published:** 2014-09-03

**Authors:** Min Wu, Martine Ben Amar

**Affiliations:** 1 Laboratoire de Physique Statistique, Ecole Normale Supérieure, UPMC Univ Paris 06, Université Paris Diderot, CNRS, 24 rue Lhomond, 75005 Paris, France; 2 Institut Universitaire de Cancérologie, Faculté de médecine, Université Pierre et Marie Curie-Paris 6, 91 Bd de l’Hôpital, 75013 Paris, France

**Keywords:** Bilayer, Finite elasticity, Volumetric growth, Contraction

## Abstract

Wound healing studies both in vitro and in vivo have received a lot of attention recently. In vivo wound healing is a multi-step process involving physiological factors such as fibrinogen forming the clot, the infiltrated inflammatory cells, the recruited fibroblasts and the differentiated myofibroblasts as well as deposited collagens. All these actors play their roles at different times, aided by a cascade of morphogenetic agents and the result for the repair is approximatively successful but the imperfection is remained for large scars with fibrosis. Here, we want to study wound healing from the viewpoint of skin biomechanics, integrating the particular layered geometry of the skin, and the role of the neighbouring wound epidermis. After 2 days post-injury, it migrates towards the wound centre to cover the hole, the migration being coupled to proliferation at the wound border. Such a process is dominated by the skin properties which varies with ages, locations, pathologies, radiations, etc. It is also controlled by passive (actin, collagen) and active (myo-fibroblasts) fibres. We explore a growth model in finite elasticity of a bilayer surrounding a circular wound, only the interior one being proliferative and contractile. We discuss the occurrence of an irregular wound geometry generated by stresses and show quantitatively that it results from the combined effects of the stiffness, the size of the wound, eventually weakened by actin cables. Comparison of our findings is made with known observations or experiments in vivo.

## Introduction

The adult skin is made of three layers: the outermost epidermis (with a thickness of a fraction of 1 mm), a thin sheet of collagen called the basement membrane (with a thickness of several microns) and the underneath dermis (with a thickness of $\sim 1$ mm) consisting of the connective tissue, blood/lymphatic vessels, hair follicles and various glands. To retrieve the local integrity, wound healing occurs after an injury of the skin which is often deep into the dermis (Paul 1997; Gurtner et al. 2008). It is a multistep phenomenon that relies on complex multi-species cell–cell interactions, involving four sequential and overlapping stages: clotting, inflammation, re-epithelialization and remodelling. Instantly after the injury, a clot mainly composed of fibrin is formed by the coagulation of fibrinogens with the blood from damaged vessels (stage 1). It stops the further bleeding and serves as a provisional matrix infiltrated by the inflammatory cells, fibroblasts and neo-vasculatures in the following days (stage 2), and then it is transformed into the granulation tissue. Induced by the growth factors and chemotactic signals released by the cells therein, several layers of keratinocytes may either duplicate or crawl to cover the denuded wound (Haase et al. 2003), taking around 2–10 days. This is the re-epithelialization, stage 3, ending with a scar originated from the granulation tissue, which is covered by the newly formed epidermis. To retrieve the structure of the normal skin, the scar undergoes remodelling (stage 4), during which collagen fibres are continuously synthesized, cross-linked and degraded. The time span of this stage is strongly influenced by the initial scar from the earlier stages and can take for years. However, the success of wound healing is not guaranteed, encountering chronic wounds or disordered recovery such as hypertrophic scars, keloids and contractures. Even in the situation without pathological complications, usually the scar is highly visible and reconstructed in the absence of skin appendages.

Different from the adult wound healing (Redd et al. 2004), a full closure can be achieved in embryos driven by the actin cable that acts like a purse-string (Danjo and Gipson 1998) globally along the wound edge, presenting a perfect skin regeneration. Unsurprisingly, the dorsal closure during *Drosophila* morphogenesis shares the same feature and has been extensively studied, which is considered as a powerful model system for wound healing. In parallel, at the late stage of re-epithelialization in mice, the myofibroblastic contraction can bring the edges of the wound closer which reduces the size of the wound (Mawaki et al. 2007). However, for most of the adult wounds except in those loose skin areas (i.e., in the neck Ehrlich and Hunt 2012), re-epithelialization (keratinocytes proliferation and migration) is more dominant. In fact, the failure of the myofibroblast apoptosis at the end of re-epithelialization can lead to the fibrocontractive diseases (i.e., hypertrophic scars), influenced strongly by the mechanical environment (Gabbiani 2003).

Aside from the stress-induced pathological conditions, attentions have been paid on the use of the mechanical environment in the surgeries, such as the incisions in the direction of the Langer’s line 1 to facilitate better healing. Careful as it is, the wound may still be covered with thick scar tissue or sometimes become chronic (Mustoe et al. 2006; Greaves et al. 2013). On the contrary to those by surgical incisions and punctures with regular geometry, most of the human wounds, especially wounds with large areas are caused by accidents, resulting in all kinds of combinations of the related regional geometry and the mechanical environment. Robust strategies have been evolved to cope with these discrepancies to restore the stress-related homeostasis of the skin. The scar tissue may well be the sacrifice for this purpose. Skin regions exposed with high level of stresses are associated with more visible scars and fibrosis (Rei 2008). It has been recognized that the mechanical stresses play a pivotal role in this process and it was shown that implementing a polymer shield on the skin to reduce the local skin tension results in a lighter scar (less fibrosis) (Gurtner et al. 2011). However, this process also reduces the area (volume) of the wound, so the detailed mechanism of the interplay between the stresses, the involved biological pathways and the geometry remains to be elucidated in complementary to the molecular-based theory. Nevertheless, understanding the role of the stress distribution during the re-epithelialization will shed a light on the comprehension and manipulation of the mechano-transduction pathways involved. The modulation of the stress distribution can be translated into therapeutical strategies which traditionally target on the biochemistry.

**Fig. 1:**
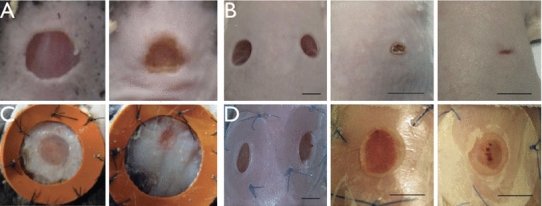
**a** Snapshots taken from day 0 and 3 from the wound closing process after a 5-mm-diameter punch biopsy (removing epidermis, basement membrane and the dermis, the same in **b**–**d**) on the normal skin of wild-type mice (Nassar et al. (2012), copyright with permission). The undulation (*right*) of the wound margin corresponds to the wavenumber $m=3$. **b** Snapshots taken from the closing process at day 0, 7 and 14 after 5-mm-diameter punches on the normal skin of wild-type mice (Wang et al. (2013), copyright with permission). Irregularities of the wound are observed and announced by authors of (Wang et al. 2013). **c** Snapshots taken at day 4 and 6 during a splinted re-epithelialization process started from a 6-mm circular wound (Nauta et al. (2013), copyright with permission). At day 6 (*right*), the wound is highly deformed from a circle (*left*). **d** Snapshots taken from the splinted re-epithelialization process at day 0, 7 and 14 after 5-mm-diameter punches on the normal skin of wild-type mice (Wang et al. (2013), copyright with permission). Without contraction, the healing process is slow, however, the border irregularity is still obvious at day 14 (*right*)

Focusing on re-epithelialization in vitro, several groups (Carrier et al. 2008; Trepat et al. 2009; Anon et al. 2012; Kim et al. 2013) have studied the collective behaviour of cells through the migration, the generation of forces, the re-organization of the cell cytoskeleton at the wound margin but within a single layer on solid substrates, indicating a non-local and robust hole-filling strategy. Among the geometries considered in vitro, the half plane and two facing half planes of cell sheets were studied. Recently, the circular geometry has received more attention which has also been carried out in the studies in vivo [i.e., Mawaki et al. (2007); Nassar et al. (2012); Saher et al. (2011); Nauta et al. (2013); Wang et al. (2013)] (Fig. 1). In vivo, the mammalian wound from the experiment is usually implemented by the punch biopsy where a cylindrical blade removes a circular part of the skin (i.e., Fig.1). In fact, the punch biopsy is also applied to excise skin tumours suspicious of malignancy, such as melanomas. For the wound healing in skin, abundant chemical signals have been released by the inflammatory cells in the granulation tissue, which is in contrast to the in vitro experiments (Trepat et al. 2009; Anon et al. 2012; Kim et al. 2013) where the environment of the cells from the same species is well controlled.

In our recent theoretical work (Ben Amar and Wu 2014), we have paid special attention to the epithelium frontier driven by chemotaxis (cell crawling) from a circular wound. This model has described the migration of the epithelium surrounding the wound, in the direction of the injury, driven by the concentration gradient of chemoattractants. The conclusion is that the circularity is broken immediately for realistic wound sizes (in the order of centimetres) as chemotaxis proceeds. It suggests that the instability of one wound border can be induced by active crawling alone. Interestingly, many of the in vivo experiments on mice wound healing also present an undulation of the wound border (Mawaki et al. 2007; Nassar et al. 2012; Saher et al. 2011; Nauta et al. 2013; Wang et al. 2013) while those for the pigs are usually circular (or elliptical due to the pre-stretch of the skin) (Hinrichsen et al. 1998). The different behaviours in the symmetry breaking of the wound and the displacement remodelling throughout the surrounding skin tissue cannot be explained from Ben Amar and Wu (2014): It may be a result of the stresses as well as the biomechanics properties of the skin.

Growth is known to generate residual stresses when the surrounding space is not compatible. The growth-induced shape deformation (instability) is known as one of the driving forces during early development of animals (Ben Amar and Goriely 2005; Ciarletta and Ben Amar 2012b; Ben Amar and Jia 2013) in concert with the mechano-transduction. When it comes to a large open wound, the proliferation of keratinocytes becomes critical during re-epithelialization. However, adult wound healing is a (re-)generation surrounded by the fully developed and well- constituted tissue. Supported but also constrained by the neighbouring tissues, the newly formed epithelial sheet expands inward and residual stresses are expected despite the migration of the cells at the very front of the proliferation region. The irregularities of the wound margin involving the remodelling of the neighbouring skin (Fig. 1c,d) may be induced by the residual stresses. It is suggested that the mechanical environment plays an equally important role in achieving true regeneration and alleviating pathological conditions (Wong et al. 2011). The irregularity of the wound margin may further disturb the stress distribution and thus requires a higher level of the modulation in the stages of the scar formation and remodelling to re-establish the homeostasis of the mechanical environment (Eckes and Krieg 2004).

In the mechanical point of view, mathematical models have been developed for dorsal closure during *Drosophila* morphogenesis, against the experiments, considering both the elastic stresses generated by the epidermis and the amnioserosa along the wound edge as well as the contractile forces generated by the actin cables (purse-string) (Hutson et al. 2003; Layton et al. 2009). Starting with an eye-shape open area, it is suggested that both kinds of forces are critical to achieve the final closure. In (Sadovsky and Wan 2007), a theoretical elasto-dynamical model has been developed, being an extension from Sherratt et al. (1992) and considering the circular wound in embryonic mammalian skin. Driven by the active stresses from the actin cables, the size of the wound decreases as a function of time starting with a quasi-equilibrium due to the reorganization of the fibres. In Taber (2009), a general theory was developed describing the mechanical feedback to the growth rate during morphogenesis and was applied to the embryonic wound healing in the circular geometry with tension. Very recently, a computational model has been developed in Wyczalkowski et al. (2013) considering both circular and elliptical geometries, with experiments, displaying a biphasic behaviour of the wound closure first promoted by the actomyosin isotropic contraction within a thick ring and later by a circumferential contraction within a thinner ring. However, for the mouse models, by the contraction of myofibroblasts which is responsible for 80 percents of the wound area reduction (Mawaki et al. 2007), the initial circularity can be lost (Fig. 1a, b) compared to the embryonic wound healing (Wyczalkowski et al. 2013).

**Fig. 2:**
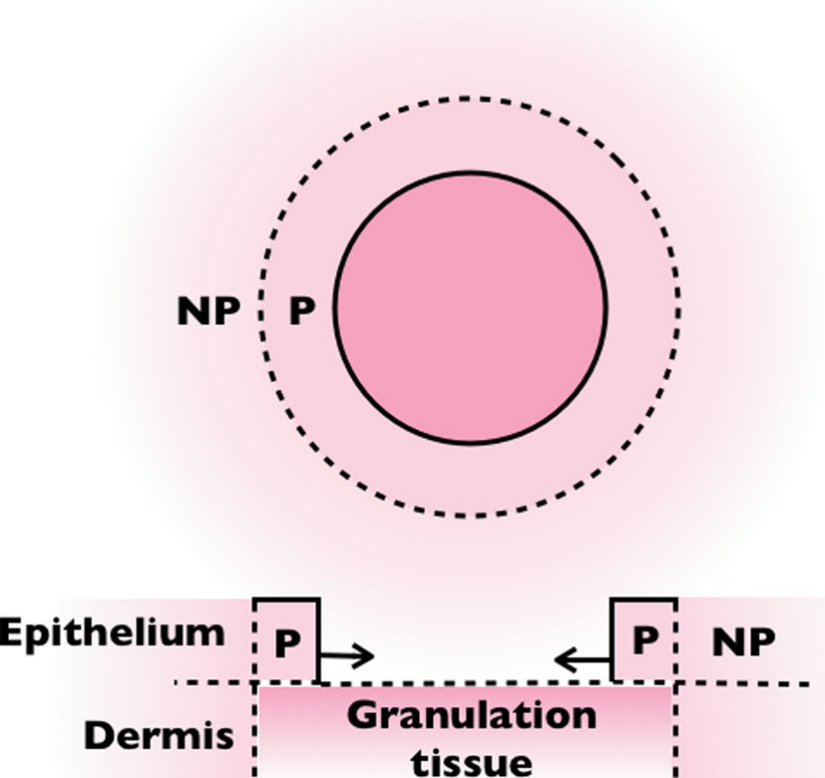
The scheme of the model. “**P**” (“**NP**”) stands for the proliferating layer (non-proliferating neighbouring epidermis). The model is $2D$ due to the assumption that there is no displacement in the epithelium-dermis (*vertical*) direction. The re-epithelialization contributes to the expansion in the direction of the wound centre (*solid arrows*)

It is why in this paper, we aim to understand the re-epithelialization and the contraction in vivo during wound healing in the framework of finite elasticity. We simulate the wound in a bilayer model (Filas et al. 2012, 2013; Moulton and Goriely 2011; Cao et al. 2012) where we consider that the growth occurs closely at the border and the newly formed tissue has different properties from that of the skin surrounding the wound (Fig. 2). Many biological tissues concern two or more layers with different mechanical properties and growths, which are more challenging to tackle compared to single layer problems. Here, the displacement of the two layers can be solved fully analytically at least in the linear order in both layers under the re-epithelialization and the myofibroblastic contraction, based on the method developed in previous works (Ben Amar and Ciarletta 2010; Ciarletta and Ben Amar 2012) imposing tissue incompressibility with a stream function. In the following, we first present the model in Sect. 2 where the formulation in finite elasticity and the variational method aided by a stream function is given. Then, we present the results in Sect. 3 exploring the effects of the stiffness of the skin, the size of the wound, the actin cables and the myofibroblastic contractions. The calculations of the stresses in the skin surrounding the wound border are provided by the model in correspondence to the deformed configuration. Finally, in Sect. 4, we make the conclusion of our major results and discuss them with the complexity of the mechanics of the skin.

## Model description

### Formulation

During re-epithelialization, the epithelial cells behind the wound border proliferate and migrate within a distance of several cell-lengths while those away from the front are connected to the surrounding skin with full integrity. Although without proliferation, it is usually observed that the neighbouring skin is also remodelled passively to maintain the continuity between the newly formed epidermis and the surrounding tissue. As a result, we define a bilayer model in $2D$ assuming that there is no thickness variation (in the epidermal–dermal dimension). Although there is a possible effect from the adhesion between the epithelial cells and the underlying granulation tissue, specially closed to the border, the adhesion is observed to be transient to facilitate cell crawling (Carrier et al. 2008), where the proliferating layer is not fully attached to the lower layer at all times. As a result, we discard the interaction between the epidermis and the dermis/granulation tissue. We consider the mass left by a hole with the circular geometry in the reference polar coordinate $(R,\varTheta )\in [R_0,\infty )\times [0,2\pi )$. The inner layer $(R,\varTheta )\in [R_0,R_0+d)\times [0,2\pi )$ grows, while the outer layer $(R,\varTheta )\in [R_0+d,\infty )\times [0,2\pi )$ extending to infinity is without growth and free of stress. In embryonic wound healing, it is strongly suggested that, the mechanical environment can feedback to the growth (contraction) rate via mechano-transduction in a spatial–temporal manner (Taber 2009). This may be one explanation for the maintenance of the circularity of the embryonic wound healing. However, the mechano-tranduction during the adult wound healing is far from clear.

**Fig. 3:**
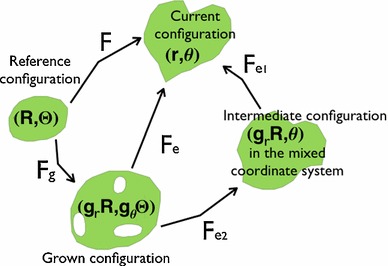
The schematic description of the growth in elasticity. $(g_rR,g_\theta \varTheta )$ represents the grown configuration in the reference coordinate system $(R,\varTheta )$ after the mapping $\mathbf{F_g}$ while $(g_rR,\theta )$ represents the intermediate configuration in the mixed coordinate system $(R,\theta )$ after the mapping $\mathbf{F}_{\mathbf{e2}}$

For the sake of simplicity, we model the growth accumulated over time by the isotropic growth $g_r$ (considering the orientation of the healthy cell mitosis is nearly random), as a step function in space, instead of a continuous transition between the proliferating and non-proliferating region. Anisotropy can be introduced by the myofibroblastic contractions. At the late stage of the re-epithelialization, the myofibroblasts derived from the fibroblasts distribute and orient along the border of the wound. Different from fibres which inactively reinforce the skin, myofibroblasts contract and contribute to a tremendous wound area reduction for loose skins. Here, we model this active contraction by the growth anisotropy $g_\theta$. $g_\theta <1$ has been accounted for cytoskeletal and actomyosin contraction in early brain development (Filas et al. 2012, 2013) and the embryonic wound healing (Taber 2009; Wyczalkowski et al. 2013). The volumetric growth (Rodriguez et al. 1994) is then modelled in finite elasticity, using the multiplicative decomposition of the deformation gradient $\mathbf{F}={\frac{\partial {\mathbf{x}}}{\partial {\mathbf{X}}}}=\mathbf{F}_\mathbf{e}\mathbf{F}_\mathbf{g}$ (Fig. 3), where $\mathbf{F_g}$ is the growth tensor $\text {diag}(g_r,g_r g_\theta )$ and $\mathbf{F_e}$ is the elastic deformation gradient from the virtual stress-free grown configuration $(g_r R, g_\theta \varTheta )$ to the current configuration $(r(R,\varTheta ),\theta (R,\varTheta ))$. The local volume increase is given by $J=\text {det}\, {\mathbf{F_g}}=g_r^2g_\theta$ for $R_0\le R<R_0+d$. The neighbouring skin is without growth thus $J=1$ when $R\ge R_0+d$. The growth-induced stress in the stress-free configuration can be obtained by the definition of the nominal stress tensor (Ben Amar and Goriely 2005)

1 $$\begin{aligned} \mathbf{S}=\frac{\partial {\varvec{\varPsi }}}{\partial {\mathbf{F}}}=J\mathbf{F_g^{-1}}\left( \frac{\partial {\varvec{\varPsi }}}{\partial {\mathbf{F}_\mathbf{e}}}-q\mathbf{F_e^{-1}}\right) \end{aligned}$$

given the strain energy function ${\varvec{\varPsi }}$ where $q$ is the Lagrange multiplier to ensure incompressibility. The human skin is almost incompressible, and this constraint gives an advantage to the analysis by introducing a non-linear partial differential equation (PDE) ($\det \mathbf{F}_\mathbf{e}=1$) for $r(R,\varTheta )$ and $\theta (R,\varTheta )$. However, the analysis becomes complicated coupled with finite elasticity. In this paper, we use the method developed in earlier works (Ben Amar and Ciarletta 2010; Ciarletta and Ben Amar 2012; Carroll 2004; Ciarletta and Ben Amar 2012a, b; Ben Amar and Jia 2013) which satisfies the incompressibility automatically by introducing the stream function $\varPhi (R,\theta )$ in $2D$. At first, we decompose the elastic deformation gradient in the mixed coordinate system $(R,\theta )$ by ${\mathbf{F_e=F_{e1}F_{e2}}}$ (See Fig. 3) where

2 $$\begin{aligned} {\mathbf{F_{e1}}}=\left[ \begin{array}{l@{\quad }l} \frac{r_{,R}}{g_r} &{}\frac{r_{,\theta }}{g_r R}\\ 0&{}\frac{r}{g_r R} \end{array} \right] ;\text { } {\mathbf{F}_{\mathbf{e2}}}=\left[ \begin{array}{l@{\quad }l} 1&{}0\\ -\frac{R\varTheta _{,R}}{\varTheta _{,\theta }}&{}\frac{1}{g_\theta \varTheta {,_\theta }} \end{array} \right] . \end{aligned}$$

under the restriction where $\varTheta _{,\theta }\ne 0, \pm \infty$. Then, the incompressibility $1=\det \mathbf{F}_\mathbf{e}=\det {\mathbf{F_{e1}}}\det {\mathbf{F}_{\mathbf{e2}}}=\frac{(r^2)_{,R}}{2JR \varTheta _{,\theta }}$ can be satisfied automatically by introducing $\varPhi (R,\theta )$ where

3 $$\begin{aligned} r=\sqrt{2\varPhi _{,\theta }}\,\,\hbox { and }\,\, \varTheta =\frac{\varPhi _{,R}}{J R} \end{aligned}$$

with $\varPhi _{,R\theta }\ne 0, \pm \infty$. At last, the isochoric (volume-preserving) deformation $\mathbf{F}_\mathbf{e}$ can be rewritten in the form of $\varPhi (R,\theta )$ by:

4 $$\begin{aligned} {\mathbf{F_{e}}}=\left[ \begin{array}{l@{\quad }l} \frac{\left( \varPhi _{,R\theta }-\frac{\varPhi _{,\theta \theta }(\varPhi _{,RR}-\varPhi _{,R}/R)}{\varPhi _{,R\theta }}\right) }{g_r\sqrt{2\varPhi _{,\theta }}} &{} \frac{g_r}{\sqrt{2\varPhi _{,\theta }}}\frac{\varPhi _{\theta \theta }}{\varPhi _{,R\theta }}\\ -\frac{\sqrt{2\varPhi _{,\theta }}}{g_r}\frac{(\varPhi _{,RR}-\varPhi _{,R}/R)}{\varPhi _{,R\theta }} &{} \frac{g_r \sqrt{2\varPhi _{,\theta }}}{{ \varPhi _{,R\theta }}} \end{array} \right] . \end{aligned}$$

### The variational method

The mechanical response of the skin is tightly related to the collagen fibres. The epidermis is anchored to the dermis across the basement membrane via collagen type VII, while the dermis is mainly composed of collagen type I, III and V and fibre-associated macromolecules. In uni-directional tension, due to the fibres, the stress–strain response of the skin is multiphase by first presenting low modulus, then increasing linearly and eventually yielding (Silver et al. 2003), which can be condensed and shifted by bi-directional stretch. Also, the skin is pre-stressed (in tension) along the Langer’s line (Annaidh et al. 2012) which complicates the true stress–strain law in vivo and also during wound healing. Nevertheless, the property of the newly formed epidermis and the granulation tissue underneath is rarely studied in biomechanical measurements (probably not practical). For the sake of simplicity, we consider the Neo-Hookean material as an approximation of both the surrounding skin and the proliferating tissue with the energy density in the coordinate system $(R,\varTheta )$:

5 $$\begin{aligned} \varPsi (\varPhi )=c_iJ(\mathbf{F}_\mathbf{e}^T\mathbf{F}_\mathbf{e}:\mathbf{I}-2) \end{aligned}$$

with different elastic modulus $c_i=c_{in}$ for the growing tissue and $c_i=c_{out}$ for the neighbouring skin. The total bulk potential energy is then:

6 $$\begin{aligned} \varPi (\varPhi )&= \int ^{\infty }_{R_0} \int ^{2\pi }_{0} \varPsi (\varPhi )\det ({\mathbf{F}_{\mathbf{e2}}})^{-1}R d R d\theta \nonumber \\&= \int ^{\infty }_{R_0} \int ^{2\pi }_{0} \varPsi (\varPhi )\frac{\varPhi _{,R\theta }}{J} d R d\theta \nonumber \\&= \int ^{\infty }_{R_0} \int ^{2\pi }_{0} \overline{\varPi }(\varPhi )d R d\theta \end{aligned}$$

in the mixed coordinate system $(R,\theta )$ where

7 $$\begin{aligned} \overline{\varPi }(\varPhi )&= c_i{\varPhi _{,R\theta }}\left[ \frac{\varPhi _{,\theta \theta }^2 J}{2g_\theta \varPhi _{,\theta } \varPhi _{,R\theta }^2}\right. \nonumber \\&\left. +\frac{g_\theta (\varPhi _{,\theta \theta } (\varPhi _{,R}-\varPhi _{,RR} R)+\varPhi _{,R\theta }^2 R)^2}{2\varPhi _{,\theta } \varPhi _{,R\theta }^2 R^2 J}\right. \nonumber \\&\left. +\frac{2 g_\theta \varPhi _{,\theta } (\varPhi _{,R}-\varPhi _{,RR} R)^2}{\varPhi _{,R\theta }^2 R^2 J}+\frac{2 \varPhi _{,\theta } J}{g_\theta \varPhi _{,R\theta }^2}-2\right] . \end{aligned}$$

Although the coordinated “purse string” effect during embryonic wound healing is lost in adults, the actin cables at the wound edge are observed experimentally (Cochet-Escartin et al. 2014) in vitro and we consider it as the surface tension at the wound edge ($R_{\text{ in }}=R_0$). At the interface ($R_{\text{ out }}=R_0+d$) between the growing epithelium and the non-proliferating skin defined in the model, we also consider a surface tension reminiscent of the regularity of the sharp transition of $g_r$ at the interface, to ensure mechanical equilibrium mathematically. As a result, we consider the surface energy on each interface $R_i$

8 $$\begin{aligned} \varGamma _i(\varPhi )= \int ^{2\pi }_{0} \overline{\varGamma _i}d\theta ]_{R=R_i} \end{aligned}$$

where

9 $$\begin{aligned} \overline{\varGamma _i}=\gamma _i\sqrt{\frac{\varPhi _{\theta \theta }^2}{2\varPhi _{\theta }}+2 \varPhi _{\theta }} \end{aligned}$$

where $i$ stands either for the index of the wound edge or of the neighbour skin interface. All together, we define the problem in a variational formulation with $\varPhi$:

10 $$\begin{aligned} \delta \varPi (\varPhi )+\Sigma _i\delta \varGamma _i(\varPhi )=0 \end{aligned}$$

which gives the following Euler–Lagrange equation in the bulk:

11 $$\begin{aligned}&-\left( \frac{\partial {\overline{\varPi }}}{\partial {\varPhi _{,\theta }}}\right) _{,\theta }-\left( \frac{\partial {\overline{\varPi }}}{\partial {\varPhi _{,R}}}\right) _{,R} +\left( \frac{\partial {\overline{\varPi }}}{\partial {\varPhi _{,R\theta }}}\right) _{,R\theta }\nonumber \\&+\left( \frac{\partial {\overline{\varPi }}}{\partial {\varPhi _{,\theta \theta }}}\right) _{,\theta \theta }+\left( \frac{\partial {\overline{\varPi }}}{\partial {\varPhi _{,RR}}}\right) _{,RR}=0 \end{aligned}$$

and two boundary conditions for the free wound edge:

12 $$\begin{aligned} c_{\text{ in }}\left[ \frac{\partial {\overline{\varPi }}}{\partial {\varPhi _{,RR}}} \right] =0 \end{aligned}$$

and

13 $$\begin{aligned}&-c_{\text{ in }}\left[ \frac{\partial {\overline{\varPi }}}{\partial {\varPhi _{,R}}}-\left( \frac{\partial {\overline{\varPi }}}{\partial {\varPhi _{,RR}}}\right) _{,R}-\left( \frac{\partial {\overline{\varPi }}}{\partial {\varPhi _{,R\theta }}}\right) _{,\theta }\right] \nonumber \\&+\gamma _{\text{ in }}\left[ \left( \frac{\partial {\overline{C}}}{\partial {\varPhi _{,\theta \theta }}}\right) _{\theta \theta }-\left( \frac{\partial {\overline{C}}}{\partial {\varPhi _{,\theta }}}\right) _{\theta }\right] =0. \end{aligned}$$

Equations (12) and (13) are equivalent to the cancellation of the normal and the shear stress in the mixed coordinate, which is demonstrated in Ben Amar and Ciarletta (2010); Ciarletta and Ben Amar (2012). For the two boundary conditions of the neighbour skin interface, we account for the continuity of the stresses in the mixed coordinate system:

14 $$\begin{aligned} c_{\text{ in }}\left[ \frac{\partial {\overline{\varPi }}}{\partial {\varPhi _{,RR}}} \right] =c_{\text{ out }}\left[ \frac{\partial {\overline{\varPi }}}{\partial {\varPhi _{,RR}}} \right] \end{aligned}$$

and

15 $$\begin{aligned}&-c_{\text{ in }}\left[ \frac{\partial {\overline{\varPi }}}{\partial {\varPhi _{,R}}}-\left( \frac{\partial {\overline{\varPi }}}{\partial {\varPhi _{,RR}}}\right) _{,R}-\left( \frac{\partial {\overline{\varPi }}}{\partial {\varPhi _{,R\theta }}}\right) _{,\theta }\right] \nonumber \\&+\gamma _{\text{ in }}\left[ \left( \frac{\partial {\overline{C}}}{\partial {\varPhi _{,\theta \theta }}}\right) _{\theta \theta }-\left( \frac{\partial {\overline{C}}}{\partial {\varPhi _{,\theta }}}\right) _{\theta }\right] =\nonumber \\&-c_{\text{ out }}\left[ \frac{\partial {\overline{\varPi }}}{\partial {\varPhi _{,R}}}-\left( \frac{\partial {\overline{\varPi }}}{\partial {\varPhi _{,RR}}}\right) _{,R}-\left( \frac{\partial {\overline{\varPi }}}{\partial {\varPhi _{,R\theta }}}\right) _{,\theta }\right] \nonumber \\&+\gamma _{\text{ out }}\left[ \left( \frac{\partial {\overline{C}}}{\partial {\varPhi _{,\theta \theta }}}\right) _{\theta \theta }-\left( \frac{\partial {\overline{C}}}{\partial {\varPhi _{,\theta }}}\right) _{\theta }\right] \end{aligned}$$

where in the rest of the paper we rescaled $c_{\text{ in }},\, c_{\text{ out }},\, \gamma _{\text{ in }}$ and $\gamma _{\text{ out }}$ by $c_{\text{ in }}$.

## Results

For a simple solution $\varTheta = \theta$ in Eq. (3), we have

16 $$\begin{aligned} \varPhi _{,R}=JR\theta \ \text { thus }\ \varPhi (R,\theta )=\frac{J(R^2+a)\theta }{2} \end{aligned}$$

satisfying the bulk Eq. (11) and the boundary conditions Eqs. (12) and (13). Taking $r(R_0+d)=R_0+d$ due to the incompressibility of the non-proliferating neighbouring region in the zero order, we have $a=(\frac{1}{J}-1)(R_0+d)^2$. While Eqs. (12) and (13) provide only the equivalent boundary conditions for the stresses in the mixed coordinate for the boundary value problem, Eq. (1) gives the exact radial and hoop stresses

17 $$\begin{aligned} S_{RR}=\frac{\sqrt{J} \left( g_\theta R^2-\left( a+R^2\right) q\right) }{R \sqrt{ a+R^2}} \end{aligned}$$

and

18 $$\begin{aligned} S_{\varTheta \varTheta }=\frac{\sqrt{J} \left( a+R^2-g_\theta R^2 q\right) }{g_\theta R \sqrt{a+R^2}}, \end{aligned}$$

defined in the stress-free configuration. The Lagrange multiplier $q$ reads:

19 $$\begin{aligned} q=\frac{1}{2} g_\theta \left( \log \left( a+R^2\right) -\frac{a}{a+R^2}\right) -\frac{\log (R)}{g_\theta }+C, \end{aligned}$$

and ensures incompressibility in the circular geometry, while $C$ is given by

20 $$\begin{aligned} C=\frac{g_\theta ^2 \left( -D \log D+a+2 R_0^2\right) +2 D \log (R_0)}{2 g_\theta D} \end{aligned}$$

in which

21 $$\begin{aligned} D=a+R_0^2 \end{aligned}$$

to cancel the radial stress $S_{RR}$ at the free boundary. When there is no net growth ($J=1$), both the wound and the neighbouring tissue is stress-free. However, the circumferential contraction contributes to tensile stresses (positive $S_{RR}$ and $S_{\varTheta \varTheta }$). The stresses $S_{RR}$ and $S_{\varTheta \varTheta }$ at the interface $R_0+d$ are plotted as a function of the local curvature $d/R_0$ in Fig. 4. With proliferation $J>1$, as expected, the stresses become compressive even for the isotropic growth (i.e., $g_\theta =1$), shown by black curves in Fig. 4. Slight contractions along the circumferential direction contribute to tensile forces in all directions ($g_\theta <1$, red and cyan curves in Fig. 4), which is particular for the wound healing process. For example, when the growth is not to fill a hole, but to expand from an inner region (i.e., the disk expansion), $g_\theta <1$ contributes to compression in $S_{RR}$ in the disk and a transition from compression to tension in $S_{\varTheta \varTheta }$. This suggests that the combination of the myofibroblast contraction and the hole-filling geometry contributes to a tensile stress in both the proliferation region and the neighbouring tissue. To further investigate whether the loss of circularity can be induced simply by the growth or contraction in the proliferating layer, let us now consider $\varPhi$ with a small perturbation of the amplitude $\epsilon$ by:

**Fig. 4:**
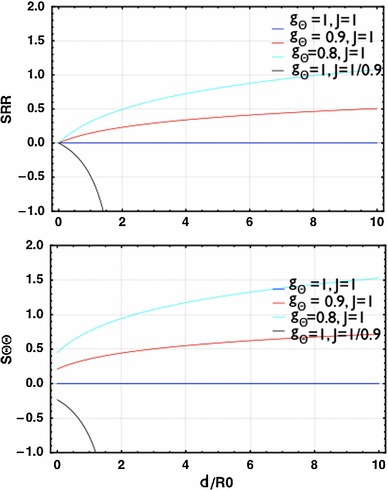
The radial ($S_{RR}$) and hoop ($S_{\varTheta \varTheta }$) stresses at the interface between the proliferating and non-proliferating region. There are no stresses when there is neither growth ($J=1$) nor contraction ($g_\varTheta =1$, *blue curves*). The compression occurs in both directions when there is only volumetric growth ($J=1/0.9$ and $g_\varTheta =1$, *black curves*). The tension occurs in both directions when there is only circumferential contraction ($g_\varTheta =0.8,0.9$ and $J=1$, cyan and red curves)

22 $$\begin{aligned} \varPhi (R,\theta )&= \frac{J(R^2+a)\theta }{2}\nonumber \\&+\epsilon \sqrt{J(R^2+a)} u(\sqrt{J(R^2+a)})\cos (m\theta ) \end{aligned}$$

and the corresponding

23 $$\begin{aligned} r(R,\theta )=\sqrt{J(R^2+a)}-m \epsilon u({\sqrt{J(R^2+a)}})\sin (m\theta ). \end{aligned}$$

where $m$ is the wavenumber. Substituting Eq. (22) into Eqs. (11), (12) and (13), and collecting the first order in $\epsilon$, we have the following Euler–Lagrange equation for $u(r)$ in the bulk:

24 $$\begin{aligned}&J (-1+m^2) R^2 (m^2 r^4+J g_\theta ^2 R^2 (-4 r^2+3 J R^2)) u(r)\nonumber \\&+r ((2 m^2 r^6-3 J m^2 r^4 R^2-2 J^2 g_\theta ^2 (2+m^2) r^2 R^4\nonumber \\&+J^3 g_\theta ^2 (3+m^2) R^6) u^\prime (r)+J r R^2 (-(m^2 r^4-8 J g_\theta ^2 r^2 R^2\nonumber \\&+J^2 g_\theta ^2 (3+m^2) R^4) u^{\prime \prime }(r)\!+\!J g_\theta ^2 r R^2 (2 (2 r^2+J R^2) u^{(3)}(r)\nonumber \\&+J r R^2 u^{(4)}(r))))=0. \end{aligned}$$

At the free boundary, $r=\sqrt{JR_0^2+(1-J)(R_0+d)^2}$, we account for zeros of the stresses in the mixed coordinate system:

25 $$\begin{aligned} S_S=(-1 + m^2) u(r) + r (u^\prime (r) + r u^{\prime \prime }(r)) \end{aligned}$$

and

26 $$\begin{aligned} S_N&= J g_\theta ^2 (-1+m^2) R^2 (-2 r^2+J R^2) u(r) \nonumber \\&+\,r ((m^2 r^4-2 J g_\theta ^2 r^2 R^2+J^2 g_\theta ^2 (1+2 m^2) R^4) u\prime (r) \nonumber \\&-\,J g_\theta ^2 r R^2 (2 (r^2+J R^2) (uu^{\prime \prime }(r)+J r R^2 u^{(3)}(r))))) \nonumber \\&-\,(\gamma _i /{2 c_i})J^2 g_\theta (-1+m^2) R^2 m^2 r u(r). \end{aligned}$$

For the interface between the proliferating and non-proliferating skin, $r=R_0+d$, we account for the continuity of the stresses $S_S$ and $S_N$ together with the continuity of the displacement:

27 $$\begin{aligned} u(r)|_-=u(r)|_+\ \text {, } \ u^{\prime }(r)|_-=u^{\prime }(r)|_+ \ \ \ \text {at}\ r=R_0+d.\nonumber \\ \end{aligned}$$

Due to the non-linearity of the coefficients in Eq. (24), it is hopeless to find the exact solution. However, under active myofibroblastic contraction without additional cell proliferation ($J=g_r^2g_\theta =1$), that is, the tissue is stressed due to the contraction but not to the volumetric growth, we have the solution for the displacement:

28 $$\begin{aligned}&b_1 r^{-\frac{\sqrt{g_\theta ^2 \left( m^2+2\right) +m \left( m+\sqrt{\left( g_\theta ^2-1\right) ^2 m^2+8 \left( g_\theta ^4+g_\theta ^2\right) }\right) }}{\sqrt{2} g_\theta }} \nonumber \\&+b_2 r^{-\frac{\sqrt{g_\theta ^2 \left( m^2+2\right) +m \left( m-\sqrt{\left( g_\theta ^2-1\right) ^2 m^2+8 \left( g_\theta ^4+g_\theta ^2\right) }\right) }}{\sqrt{2} g_\theta }}\nonumber \\&+b_3 r^{\frac{\sqrt{g_\theta ^2 \left( m^2+2\right) +m \left( m+\sqrt{\left( g_\theta ^2-1\right) ^2 m^2+8 \left( g_\theta ^4+g_\theta ^2\right) }\right) }}{\sqrt{2} g_\theta }}\nonumber \\&+b_4 r^{\frac{\sqrt{g_\theta ^2 \left( m^2+2\right) +m \left( m-\sqrt{\left( g_\theta ^2-1\right) ^2 m^2+8 \left( g_\theta ^4+g_\theta ^2\right) }\right) }}{\sqrt{2} g_\theta }} \end{aligned}$$

for arbitrary $g_\theta$ and thus

29 $$\begin{aligned} u_{nogr}(r)=b_1 r^{-m-1}+b_2 r^{1-m} \end{aligned}$$

in the non-proliferating skin neighbourhood without contraction. Eq. (29) is simplified after removing $$ b_3 r^{m+1}+ b_4 r^{m-1}$$ due to the regularity at infinity (in the condition $$m>g_\theta $$, which stays valid in the parameter regime here). Notice that $$J=1$$ for $$g_\theta \ne 1$$ suggests that: $$g_r=1/{g_\theta }^{\frac{1}{2}}\ne 1$$ to ensure the in-plane incompressibility. This indicates that a circumferential contraction induces the elongation in the radial direction, not considering cell deaths, in contrast to (Taber 2009; Wyczalkowski et al. 2013) where the contraction can induce the thickening of the contracting layer. For more general cases where $$J=g_r^2 g_\theta \ne 1$$, the coefficient of $$u(r)$$ is associated with $$m$$ of an order of 4, we can treat Eq. (24) with WKB approximation where $$u(r)\approx u_{wkb}=F(r)\exp {(m S(r))}$$ for $$m>>1$$. In a previous work (Ciarletta and Ben Amar 2012), WKB approximation has been applied to the circular geometry and was demonstrated with good accuracy of the approximated dispersion relation with respect to the existing numerical results in biomechanics literature (Moulton and Goriely 2011). The WKB approximation is obtained as

30 $$\begin{aligned} u_{wkb}(r)=&\frac{\sqrt{(d+R_0)^2 \left( g_r^2 g_\theta -1\right) +r^2}}{\sqrt{g_\theta ^2 \left( (d+R_0)^2 \left( g_r^2 g_\theta -1\right) +r^2\right) ^2-r^4 }}\nonumber \\&\times [a_1 r^{1-m}+a_2 r^{m+1}\nonumber \\&+ a_3 \left( (d+R_0)^2 \left( g_r^2 g_\theta -1\right) +r^2\right) ^{\frac{1}{2} \left( 1-\frac{m}{g_\theta }\right) }\nonumber \\&+a_4\left( (d+R_0)^2 \left( g_r^2 g_\theta -1\right) +r^2\right) ^{\frac{1}{2} \left( 1+\frac{m}{g_\theta }\right) }].\nonumber \\ \end{aligned}$$

There are six boundary conditions, two from the stress-free condition (Eqs. (25) and (26) equal to $0$) at

31 $$\begin{aligned} r_{in}=\sqrt{JR_0^2+(1-J)(R_0+d)^2}, \end{aligned}$$

two from the continuity of stresses at $r_{out}=R_0+d$, and the last two from the continuity of the deformation:

32 $$\begin{aligned} u_{wkb}(r)=u_{nogr}(r)\ \text {and }\ u^\prime _{wkb}(r)=u\prime _{nogr}(r). \end{aligned}$$

at $r_{out}=R_0+d$. We study the involved parameters by seeking for the bifurcation curves numerically out of the linear system with six boundary conditions and six coefficients $a_{1,2,3,4}$ and $b_{1,2}$.

### Isotropic growth: re-epithelialization

**Fig. 5:**
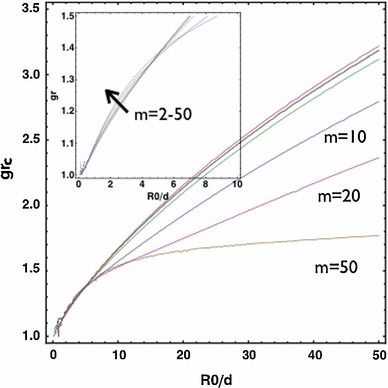
$g_{rc}$ versus $R_0/d$. For large wounds $R_0/d>>1$, higher values of the wavenumber $m$ are dominant and the critical growth $g_{rc}\sim 1.65$. For smaller wounds $R_0/d\le 10$, $g_{rc}$ decreases. There is a transition between modes near $R0/d\sim 5$

**Fig. 6:**
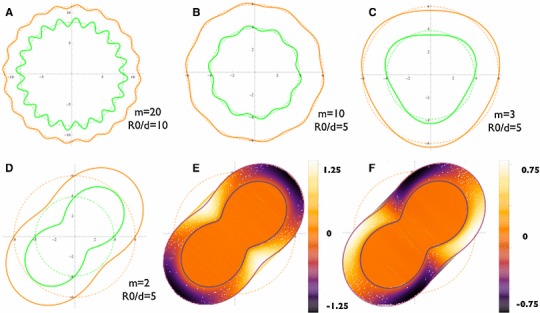
The configurations of perturbed $r=r-m \epsilon u(r)sin(m \theta )$ from the $u_{wkb}(r)$ satisfying the boundary conditions are plotted, setting an arbitrary finite value for the perturbation amplitude $\epsilon$. **a** $m=20$ when $R_0/d=20$. **b** $m=10$ when $R_0/d=5$. **c** $m=3$ when $R_0/d=5$. **d** $m=2$ when $R_0/d=5$. Both the wound border and the interface between the proliferating and non-proliferating region are deformed. For $m=2$ the normal stress ($\mathbf{e}$) and the shear stress ($\mathbf{f}$) in the first order of $\epsilon$ is plotted by the left hand side of Eqs. (12) and (13). Notice although the frontier is free of both stresses (*orange*), the undulation of the wound results in compression (*black* for the normal stress plot) or tension (*white* for the normal stress plot), and shear (*non-orange areas* for the shear stress plot) to the tissue behind the border. The stresses penetrate to the surrounding tissue due to the continuity (not shown)

Let us first analyse the stability properties during isotropic growth $g_\theta =1$ (volumetric growth only), which represent re-epithelialization considering the same skin stiffness in the neighbour tissue and the growing epithelium ($c=1$). We begin by ignoring the surface tensions ($\gamma _i$=0). Given the complexity of its implicit expression, we plot in Fig. 5 the solution of the boundary value problem in terms of the dispersion relation between the critical isotropic growth $g_{rc}$ for the instability and the size of the wound rescaled by the thickness of the growing layer $R_0/d$. For large wounds $R_0/d>>1$, higher values of the wavenumber $m$ are dominant with the critical growth $g_{rc}\sim 1.65$ thus $J\sim 2.72$. For smaller wounds $R_0/d\le 10$, the critical growth $g_{rc}$ for instability decreases and there is a transition between the critical wave numbers around $R_0/d=5$. For even smaller wounds $R_0/d\le 5$, the critical growth $g_{rc}$ decreases further and the low wave number (i.e., $m=2$) is dominant. The value of $g_{rc}$ provides a threshold for the accumulative growth for future results out of dynamical theoretical models or experimental measurements to compare. The configuration of perturbed $r=r-m \epsilon u(r)sin(m \theta )$ from the WKB solution $u_{wkb}(r)$ satisfying the boundary condition is plotted in Fig. 6 setting an arbitrary finite value for $\epsilon$. The nominal stresses in the proliferating layer have also been plotted for $m=2$ in the first order of $\epsilon$ by Eq. (1). Notice the frontier of the wound is free of normal and shear stresses at the surface (orange). However, the tissue behind the border can be under tension or compression, and shear depending on the magnitude of $\epsilon$. Such stress configurations may be compared to the photoelasticity measurements. We can anticipate $\epsilon \sim A_c\sqrt{g_r-g_{rc}}$ by a non-linear analysis where $A_c$ is in the order of $1$, where the proof is given in previous works (Ben Amar and Jia 2013; Jia and Ben Amar 2013), in which the value of $\epsilon$ is critical to select the pattern of swelling wrinkles among stripes, square or hexagons, which is not necessary in this work.

### The stiffness of the skin

**Fig. 7:**
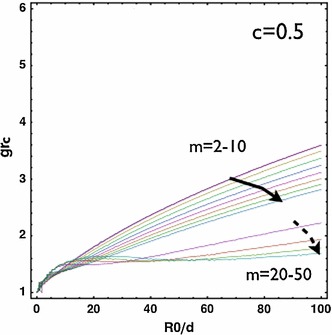
$g_{rc}$ versus $R_0/d$. For softer neighbourhood ($c=c_{\text{ out }}/c_{\text{ in }}=0.5$), the transition zone expands compared to Fig. (5)

**Fig. 8:**
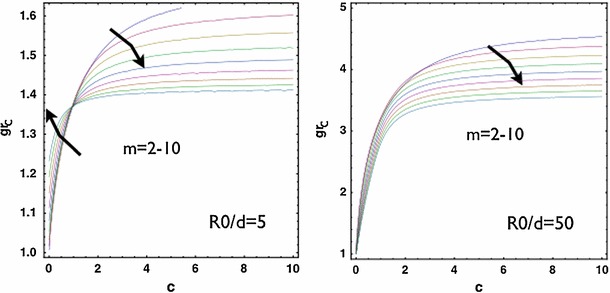
$g_{rc}$ versus $c=c_{\text{ out }}/c_{\text{ int }}$ with small wound $R_0/d=5$ (*left*) and large wound $R_0/d=50$ (*right)*. For $R_0/d=5$, there is a transition of dominant modes under the change of $c$ while for $R_0/d=50$, there is no such a transition. In both cases, the critical $g_{rc}$ increases as $c$ increases. The effect from $c$ saturates as $c$ goes beyond $c\sim 2$

The stiffness of the skin tissue differs among the subjects transcending different ages (Pawlaczyk et al. 2013; Smalls and Randall Wickett 2006) as well as in different locations (Smalls and Randall Wickett 2006) of the trauma. Also, pathological conditions and medical treatments may change the local stiffness of the skin, such as the skin tissue surrounding a tumour (i.e., melanoma) and the breast tissue after radiotherapy. For melanomas (Rofstad et al. 2006), it has been assumed that the acid mediates the solid tumour invasion by remodelling the adjacent normal tissues that allow local invasion. In recent work (Estrella et al. 2013), the peritumoral pH was measured to be low, while the lowest pH corresponds to the highest tumour invasion. On the other hand, the tumour did not invade into neighbouring regions with normal or near-normal pH. Moreover, the peritumoral extracellular matrix can

be degraded by the release and activation of proteolytic enzymes. Then, the tissue which is adjacent to the malignant skin tumour after surgical extraction may possess different stiffness from the normal skin. In this section, we consider different ratios of the stiffness between the two layers. For softer neighbourhood compared to the growing epithelium ($c=c_{\text{ out }}/c_{\text{ in }}=0.5$), the transition zone of wave numbers is shifted to larger values of $R_0/d$ and the transition zone expands (Fig. 7). For stiffer neighbourhood, the transition zone is shifted to smaller values of $R_0/d$ which indicates a higher wave number for small wounds (not shown). For a fixed wound size $R_0/d=5$, we also show the transition of wave numbers under the variation of $c=c_{\text{ out }}/c_{\text{ in }}$ in Fig. (8, left). For the case with stiffer neighbourhood, there is always a favour for large $m$ and the critical $g_{rc}\sim 1.39$. For the case with softer neighbourhood, $m=2$ is dominant and $g_{rc}$ decreases as the stiffness of the neighbourhood decreases. For large wounds (i.e., $R_0/d=50$, Fig. 8, right), large $m$ is always dominant. In both large and small wounds, $g_{rc}$ increases as the stiffness of the neighbourhood increases and eventually saturates in a fixed level ($\sim 1.4$ for small wounds and $\sim 3.5$ for large wounds). Interestingly, the skin of the pig is stiffer (Żak et al. 2011) than that of the mouse (Wang et al. 2013). The wound of the porcine skin can maintain the circularity (or the ellipticity due to pre-stretch) (Hinrichsen et al. 1998), whereas the wound of the mice’s skin shows the irregularity during re-epithelialization even when the neighbouring skin is splinted so that the mechanism of contraction is forbidden (Fig. 1, d). Experiments focusing on the same animal model across ages may further validate this result.

### The local actin cable effect

**Fig. 9:**
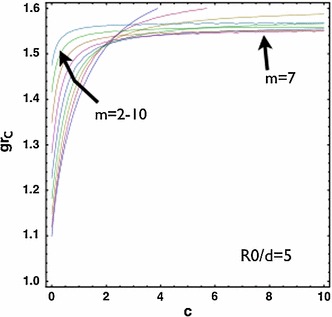
$g_{rc}$ versus $c=c_{\text{ out }}/c_{\text{ in }}$ with small wound $R_0/d=5$ considering the surface tension due to the actin cable $\gamma _{\text{ in }}/c_{\text{ in }}=1$. For $c<2,\, g_{rc}$ is slightly elevated and for $c>>2$, the dominant mode $m=7$ compared to Fig. 8, left where $m\rightarrow \infty$ is selected instead

Up to now, we see the selection of wave numbers as a result of the wound size compared to the thickness of the proliferating epithelium ($R_0/d$) and a result of the comparative stiffness $c_{\text{ out }}/c_{\text{ in }}$. Here, we show that the surface tension at $R=R_0$ due to the existence of the local actin cable, also affects the critical $m$ and $g_{rc}$ when $R_0/d$ is small. When the actin cable effect is considered at $R=R_0$, the critical mode is brought down to a finite value (i.e., $m=7$ when $\gamma _{\text{ in }}/c_{\text{ in }}=1$ in Fig. 9) in the case of stiffer neighbourhood compared to Fig. 8, (left) where the actin cable effect is not considered. In addition, the actin cable slightly elevates $g_{rc}$ in the case of softer neighbourhood. As expected physically, applying surface tension at $R=R_0+d$ has minor effect except to slightly elevate $g_{rc}$ (not shown). This also provides a possible explanation for the localization of the actin cable at the front of a wound in the aspect of evolution.

### The myofibroblastic contraction

**Fig. 10:**
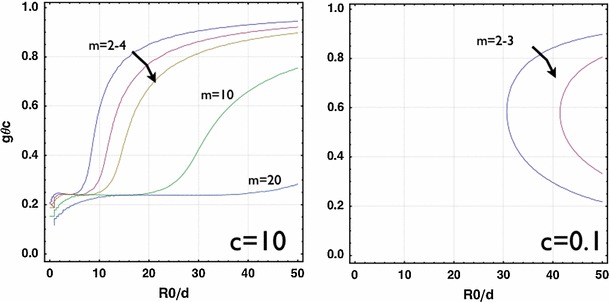
$g_{\theta c}$ versus $R_0/d$ with stiff ($c=10$) and soft ($c=0.1$) neighbourhood without net growth ($J=1$) from the exact solution. For large $R_0/d$, $g_{\theta c}$ is very close to $1$, indicating slight contraction without growth contributes to the instability. As $R_0/d$ decreases, $g_{\theta c}$ decreases further for stiff neighbourhood but disappears for soft neighbourhood. This provides an explanation for the success of “purse string” closure during embryonic wound healing due to the flexibility of the surrounding tissue

The function of myofibroblastic contraction is critical to wound healing for loose skins. During re-epithelialization, the myofibroblasts distribute and orient along the border of the wound. The contraction of myofibroblasts contributes to 80 % of the wound area reduction in mice. During embryonic wound healing, the circularity can be more or less maintained, which is not the case for mice (Fig. 1). Also, the embryonic epithelium is more soft and flexible than that of the adult, and the wound size is much smaller, so here we investigate the effect of the wound size and the stiffness of the surrounding skin to the stability. We introduce the active effect as anisotropic growth $g_\theta <1$. We consider $g_\theta <1$ and $J=1$ where there is no net proliferation but a circumferential contraction. Interestingly, for large wounds, a slight contraction contributes to the instability with $m=2$ (Fig. 10) regardless of the stiffness (both cases with $c=10$ and $c=0.1$). For small wounds, when the neighbourhood is very stiff (i.e., $c=10$), there is always a critical level of $g_{\theta c}$ with $m=2$, but for soft neighbourhood, there is no instability when the wound is moderately small (i.e., $R_0/d\le 30$ in Fig. 10, right). This result suggests that, the success of “purse string” closure during embryonic wound healing also results from the softness of the surrounding tissue combined with the smallness of the wound. Notice that the above result is from the exact analytical solution Eq. (28) for $J=1$ and arbitrary $g_\theta$. On the other hand, the WKB approximation Eq. (30) can be used to investigate the bifurcation curves for $J\ne 1$ varying $g_\theta$. However, in the parameter space close to $J=1$ and $g_\theta =1$, Eq. (30) becomes singular, which results in turning points (Bender and Orszag 1978; Ben Amar and Pomeau 1986) in this boundary value problem and brings complications for the application of the technique. The small parameter space requiring more careful analysis will be considered in future work. Despite the localized inaccuracy near $J=1$ and $g_\theta =1$, Eq.(30) can still be used to make predictions for large $J$’s far from $1$.

## Discussions

In this work, we have shown the instability of the circular wound during re-epithelialization in vivo due to the mechanical stresses introduced by the keratinocyte proliferation and the myofibroblastic contraction. The deformed pattern deviates from the circularity due to the proliferation (the dominant wave $m$). It is selected by a combination: first, the ratio between the wound size and the thickness of the proliferating layer ($R_0/d$, Fig. 5), second, the ratio of the stiffness between the neighbouring skin and the proliferating zone ($c_{\text{ out }}/c_{\text{ in }}$, Figs. 7 and 8) and third, the relative strength of the actin cable at the wound frontier ($\gamma _{\text{ in }}/c_{\text{ in }}=1$ in Fig. 9 compared with $\gamma _{\text{ in }}/c_{\text{ in }}=0$ in Fig. 8, left). Only keratinocytes at the wound margin proliferate and the thickness of the proliferation layer $d$ is fixed by the size of several cells $\sim 50 \mu m$. Then, our results provide an upper bound of the critical growth $g_{rc}$ for the instability due to the growth to occur, which is $\sim 3.5$ when the wound radius $R_0>>10\times d=0.5$ mm and the stiffness of the neighbouring tissue $c_{\text{ out }}> 2\times c_{\text{ in }}$ (Fig. 8, right). This indicates that, for stiff neighbouring skin, the circularity is able to be maintained for $g_r<g_{rc}$ at the beginning of the re-epithelialization. During the late stage of the re-epithelialization, the myofibroblasts can distribute along the wound edge, contract and bring the wound edge closer. Our results show that the myofibroblastic contraction contributes to an instantaneous instability for large wounds while for small wounds, only softer neighbouring tissue may escape from the instability.

A wound may result from the punch biopsy, in which a cylindrical instrument is used to remove a mole or a possible malignant skin tumour including a small area of normal skin around. It is usually a circle at the beginning which may further deform to an ellipse due to the pre-stretch of the skin (Fig. 1). However, the involvement of pre-stretch is beyond the scope of this study and will be considered in future as an extension from this work. In our recent work (Ben Amar and Wu 2014), where the circular wound is also considered, we have shown an instability of the circular geometry driven by chemotaxis. Interestingly, we observe that the instability occurs for large wounds varying all involved parameters with regard to the chemoattractant distribution and the migration mobility, while the circularity can be maintained only if the wound is extremely small. Similar to our conclusion in (Ben Amar and Wu 2014), here the hole-filling process from the circular geometry is always unstable if the growth $g_r$ goes beyond $3.5$, meaning the instability has to occur upon the grown thickness given by Eq. (31). As a result, the thickness for large wounds does not increase so much at the instability threshold. It is known that the epithelial layer responds to the wound first by migrating to cover the denuded area as a moving carpet, followed by cell proliferation to support the migration. While the migration and proliferation are confined near the wound edge, supported by the underneath granulation tissue, the cells in the back are connected to the surrounding skin tissue.

The differential growth between two layers, which is also the cause of buckling in the morphology of other biological processes such as the fingering of dermal–epidermal junction (Ciarletta and Ben Amar 2012b), the herringbone pattern of intestinal tissues (Ben Amar and Jia 2013), and the surface folding of oesophageal mucosa (Li et al. 2011), is highly affected by the ratio of the stiffness between the surrounding skin and the proliferating layers. In particular, for the circular wound after the biopsy punch, although the stiffness of the skin tumour (i.e., malignant melanomas) is higher than the surrounding tissue (Raveh Tilleman et al. 2004), after the extraction, it is the property of the neighbouring skin that matters. The stiffness of the skin in human depends on the position, the age (Pawlaczyk et al. 2013), the pathological condition, the radiotherapy, etc. For example, the stiffness of the skin is measured by suction techniques: On the shoulder it is smaller than that on the thigh, which is smaller than that on the calf (Smalls and Randall Wickett 2006). However, the measurement of Young’s modulus out of different techniques may vary tremendously (i.e.,$129 \pm 88$ kPa of the bulk skin in Diridollou et al. (2000), 180–286  kPa for the dermis in Li et al. (2012) and 3.6–9.3 mPa in Gennisson et al. (2004) for the dermis, all performed on the skin of the forearm).

Although there is still an uncertainty on the stiffness of the skin and a variation between cases, in some region of the body, it is postulated from our results that the effect of the stiffness saturates, being much larger than the stiffness of the granulation tissue (underneath the proliferating layer). Considering the same position individually, the elastic property of the granulation tissue is remodelled throughout the four stages, from a clot mainly composed of fibrin to a scar tissue with redundant collagen deposition. Although there is a lack of information with regard to the time dependence of the stiffness of the skin granulation tissue, it should be reinforced due to the collagen deposition from the fibroblasts. As a result, the ratio of the stiffness decreases and the undulated pattern emerges more easily during the proliferation.

On the other hand, considering the late stage of the re-epitheliazation, it is the myofibroblastic contraction that dominates the closing process for loose skin. In contrast to the early re-epitheliazation with proliferation, the smaller ratio between the neighbouring skin and the newly formed skin favours the regularity of the geometry. This corresponds to either softer skin neighbourhood or larger stiffness in the remodelling granulation tissue. Thus, the deposition of collagen in the granulation tissue actually aids the circumferential myofibroblastic contraction at the late stage. When the granulation tissue is fully covered by the new epithelium (end of the re-epithelialization), myofibroblasts are distributed everywhere underneath and their apoptosis is the start of scar formation (fibrosis). Interestingly, in Nassar et al. (2012), it has been shown the inhibition of calpains, proteins essential for the wound healing, strikingly delayed the earlier stages for the process but beneficially alleviates the excessive collagen production at the end. As a result, the inhibition of calpains at late stages may help achieving a rapid wound healing with the invisible scar in Nassar et al. (2012). Our result suggests that the positive effect of collagen deposition at the late stage of the re-epithelialization (contraction phase), and the inhibition should be launched after the contraction phase.

One major issue of the results comes from the constitutive law of the skin. The stress–strain relation of the mature skin is quite different from the relation given by Neo-Hookian materials (Silver et al. 2003). Notice if we consider more general laws (i.e., Mooney–Rivlin strain-energy function^2^ $\varPhi (I_1,I_2)=\mu _1 (I_1-2) +\mu _2 (I_2-2)$), we recover the same E-L equations in the bulk in both the zero and the first order of $\epsilon$. In the base solution where the wound remains a circle, the stresses are the same.^3^ In this case, the conclusion from our results due to the Neo-Hookian materials is unchanged, however, the collagen-induced material anisotropy may change the results. The collagen-associated behaviour of the skin (Annaidh et al. 2012; Holzapfel 2001) can be incorporated into the model without difficulties.

## Footnotes

1 Langer’s line is discovered by Karl Langer (1819–1887), revealing the natural orientation of collagen fibres in the dermis which adapts to biomechanical environment. Along the lines, the circular wounds elongate and become ellipses.

2
$$I_1=tr(\mathbf{F}_\mathbf{e}^T\mathbf{F}_\mathbf{e})$$ and $$I_2=\frac{1}{2}((tr(\mathbf{F}_\mathbf{e}^T\mathbf{F}_\mathbf{e}))^2-tr(\mathbf{F}_\mathbf{e}^T\mathbf{F}_\mathbf{e})^2)$$

3
$\mathbf{S}=J\mathbf{G}^{-1}(\frac{\partial {\varvec{\varPsi }}}{\partial {\mathbf{F}_\mathbf{e}}}-q \mathbf{F}_\mathbf{e}^{-1})=\mu _1\mathbf{F}_\mathbf{e}+\mu _2(tr(\mathbf{F}_\mathbf{e}^T\mathbf{F}_\mathbf{e})\mathbf{I}-\mathbf{F}_\mathbf{e}^T\mathbf{F}_\mathbf{e})\mathbf{F}_\mathbf{e}-q \mathbf{F}_\mathbf{e}^{-1})$ where the second term with $\mu _2$ vanishes when $\mathbf{F}_\mathbf{e}$ is diagonal.
